# Interpretable machine learning for cognitive impairment prediction in Parkinson’s disease: a multicenter validation study with SHAP analysis

**DOI:** 10.3389/fnagi.2025.1688653

**Published:** 2025-11-11

**Authors:** Ziyuan Wang, Junqiang Yan

**Affiliations:** 1 Key Laboratory of Neuromolecular Biology, The First Affiliated Hospital, College of Clinical Medicine of Henan University of Science and Technology, Luoyang, China; 2 Department of Neurology, The First Affiliated Hospital, College of Clinical Medicine of Henan University of Science and Technology, Luoyang, China

**Keywords:** Parkinson’s disease, cognitive impairment, interpretable machine learning, XGBoost, Random Forest

## Abstract

**Introduction:**

Parkinson’s disease (PD)-related cognitive impairment (PD-CI) is a common and impactful complication of PD, yet current predictive models often rely on specialized resources, lack interpretability, or have limited cross-population validation. This study aimed to develop an interpretable machine learning framework for PD-CI detection using only routine clinical data, addressing unmet needs in accessible and generalizable PD care.

**Methods:**

We analyzed 1,279 participants from the Parkinson’s Progression Markers Initiative (PPMI) as the discovery cohort and 197 patients from an independent validation cohort. PD-CI was defined by a Montreal Cognitive Assessment (MoCA) score ≤26 and Unified Parkinson’s Disease Rating Scale Part I (UPDRS-I) score ≥1. Twenty-one clinical features—encompassing hematological parameters, metabolic markers, and demographics—were preprocessed with synthetic minority over-sampling. Four machine learning models were trained and optimized via nested 5-fold cross-validation.

**Results:**

The Random Forest algorithm achieved superior performance in the discovery cohort (AUC = 0.83), outperforming CatBoost (AUC = 0.82), XGBoost (AUC = 0.79), and neural networks (AUC = 0.66). External validation of the framework preserved 71.57% accuracy. SHAP interpretability analysis identified age, neutrophil-to-lymphocyte ratio (NLR), and serum uric acid as critical predictors, revealing synergistic risk effects between elevated inflammation markers and reduced antioxidant levels.

**Discussion:**

This framework demonstrates diagnostic accuracy comparable to advanced neuroimaging while utilizing readily available clinical data, enhancing accessibility in resource-limited settings. It highlights neuroinflammation and oxidative stress as key mechanistic drivers of PD-CI, advancing pathophysiological understanding. Multicenter validation confirms the model’s robustness across ethnic populations, supporting its utility as a clinically actionable tool for PD-CI screening and monitoring.

## 1 Introduction

Parkinson’s disease (PD) is recognized as the second most prevalent neurodegenerative disorder worldwide, with cognitive impairment (PD-CI) manifesting in approximately 30% of patients at initial diagnosis (Abumalloh et al., 2024; Bloem et al., 2021). Significantly, nearly 80% of PD patients develop dementia (PDD) within 10-year follow-up periods, establishing PD-CI as the strongest predictor of caregiver burden and nursing home placement (Aarsland et al., 2017; Aarsland et al., 2021; Baiano et al., 2020).

Recent advancements in predictive modeling have explored multimodal approaches combining digital phenotyping data [e.g., wearable actigraphy patterns (Sakal et al., 2024)] and neuroimaging biomarkers [e.g., striatal dopamine transporter binding ratios (Almgren et al., 2023)]. The Shapley Additive exPlanations (SHAP) interpretability framework has emerged as a pivotal tool for overcoming the “black box” limitations inherent in conventional machine learning (ML) models. Validation studies on Parkinson’s Progression Markers Initiative (PPMI) cohorts (Parkinson Progression Marker Initiative, 2011) have demonstrated that models integrating cortical thickness measurements and clinical features achieve area under the curve (AUC) values of 0–80 in predicting cognitive decline trajectories.

Despite these developments, three critical translational barriers persist. First, current biomarker paradigms remain heavily dependent on specialized resources such as functional magnetic resonance imaging (fMRI) and epigenetic profiling. Second, limited model interpretability continues to hinder clinical adoption, particularly in non-specialized care settings. Third, existing algorithms exhibit geographical validation gaps, with fewer than 5% of published models incorporating Asian population data.

Our multicenter investigation addresses these challenges through three methodological innovations. First, we operationalize routinely accessible clinical variables, including complete blood count-derived inflammatory indices [e.g., neutrophil-to-lymphocyte ratio (NLR)] and metabolic profiles [e.g., serum uric acid (SUA)], developing cost-effective screening suitable for resource-limited settings. Second, the SHAP framework enables dynamic visualization of predictor contributions across diverse patient subgroups. Third, our validation protocol incorporates both PPMI data (*n* = 1,279; predominantly White participants) and an independent Asian cohort (*n* = 197, from China), focusing on geographical generalizability (i.e., performance across populations from different geographic regions) rather than broad transethnic evaluation. This design specifically tests whether the model can be applied beyond the White-majority PPMI cohort to an Asian PD population, addressing the aforementioned geographical validation gap.

## 2 Materials and methods

### 2.1 Participants

All data were obtained from the Parkinson’s Progression Markers Initiative (PPMI) database^1^, an ongoing international multicenter longitudinal cohort study. It should be noted that although the PPMI cohort involves participants from multiple countries, it is predominantly composed of White individuals, with relatively limited representation of Asian populations (Parkinson Progression Marker Initiative, 2011). All participating centers received approval from their respective Institutional Review Boards (IRB), with adherence to the ethical principles outlined in the Declaration of Helsinki (World Medical Association, 2013). The inclusion criteria were: (1) Diagnosis of Parkinson’s disease according to the International Parkinson and Movement Disorder Society (MDS) clinical diagnostic criteria (Postuma et al., 2015); (2) Age ≥18 years with full legal capacity; (3) Completion of baseline clinical assessments, including hematological profiles (e.g., neutrophil-to-lymphocyte ratio), neuropsychological evaluations (Montreal Cognitive Assessment, MoCA) (Nasreddine et al., 2005) and Part I of the Unified Parkinson’s Disease Rating Scale (UPDRS-I) (Zou et al., 2023); (4) Provision of legally valid informed consent (signed by the patient or legal guardian). The exclusion criteria: (1) Secondary parkinsonism (e.g., drug-induced or vascular parkinsonism); (2) Missing >10% of critical clinical data, with missingness patterns verified using Little’s Missing Completely at Random (MCAR) test; (3) Comorbid severe central nervous system disorders (e.g., history of stroke or traumatic brain injury); (4) Incomplete scale evaluations (MoCA with ≥ 2 unanswered items or UPDRS-I missing ≥ 2 domains); (5) Recent neuromodulatory interventions (e.g., deep brain stimulation) or use of anticholinergic medications (classified per Beers Criteria) within the preceding 6 months.

### 2.2 Classification framework design

PD-CI was operationalized as a composite endpoint incorporating both neuropsychological screen failures (Montreal Cognitive Assessment [MoCA] score ≤ 26) and patient-reported cognitive decline (Unified Parkinson’s Disease Rating Scale Part I [UPDRS-I] >1). This dual-criterion approach aligns with Movement Disorder Society diagnostic workflows for PD mild cognitive impairment.

### 2.3 Predictor selection protocol

Features were organized into three biologically interpretable clusters (Cui et al., 2024; Giil et al., 2019; Hosseini et al., 2023; Muñoz-Delgado et al., 2021; Nissen et al., 2019; Olsson et al., 2016; Ren et al., 2024; Sakuta et al., 2017; Shen et al., 2022; Steenland et al., 2014; van Wamelen et al., 2020):

1. Demographic characteristics: age, sex, BMI
2. Hematological biomarkers: 16 laboratory parameters spanning inflammatory indices (e.g., NLR), metabolic profiles (e.g., serum uric acid), and hematopoiesis markers (e.g., hemoglobin)
3. Neuropsychological assessments: UPDRS-I non-cognitive domains (e.g., mood, sleep disturbance) — note that the MoCA was excluded from predictors, as it was used to define the primary outcome to avoid outcome-predictor overlap and potential data leakage

SHAP guided predictor prioritization through iterative backward elimination, with Kendall’s τ correlation heatmaps verifying clinically plausible predictor-outcome relationships.

### 2.4 Predictive modeling architecture

Data from the PPMI cohort underwent rigorous preprocessing, including synthetic minority oversampling (SMOTE, k-neighbors = 5) to address class imbalance and stratified 80:20 training-test partitioning. Four machine learning architectures—Random Forest (max_depth = 12), XGBoost (learning_rate = 0.05), CatBoost (iterations = 1,000), and a fully connected neural network (3 hidden layers)—were optimized through nested 5-fold cross-validation. To prevent data leakage during cross-validation, SMOTE was applied independently within each inner training fold following this workflow: The PPMI training set (80% of total PPMI data) was split into 5 inner folds (stratified by PD-CI status); For each inner fold: a) Use 4 folds as the “inner training subset” (impute missing data if < 10% → apply SMOTE to balance classes); b) Use 1 fold as the “inner validation subset” (no SMOTE, no imputation, only observed data); Hyperparameters were tuned based on performance on the inner validation subset, and this process was repeated for all 5 inner folds to ensure no overlap between SMOTE-generated synthetic data and validation data. The outer 5-fold cross-validation followed the same logic: SMOTE was only applied to the outer training folds, and the outer test folds were used for unbiased performance evaluation without any oversampling. Bayesian hyperparameter tuning balanced model complexity with generalizability, while permutation importance analysis safeguarded against overfitting. The validation subset served dual purposes: interim performance monitoring during development and final unbiased evaluation of clinical deployment potential.

### 2.5 Geographic generalizability assessment

External validation leveraged a prospective cohort of 197 Parkinson’s disease patients from First Affiliated Hospital of Henan University of Science and Technology (FAHHAUST) from 2020 to 2024, maintaining protocol alignment with PPMI inclusion/exclusion criteria. This independent Asian cohort enabled quantification of model transportability across ethnic and healthcare system boundaries. All participants provided written informed consent under Institutional Review Board approval (Id:2024-496).

### 2.6 Model evaluation framework

Diagnostic performance was quantified through receiver operating characteristic analysis, with area under the curve (AUC) interpretation following NIH Biomarker Working Group guidelines: 0.85–0.93 (excellent), 0.75–0.84 (clinically acceptable), and 0.65–0.74 (research-grade). Additional metrics include accuracy, AUC, recall, accuracy, and F1 scores. All analyses and adjustments are carefully carried out under the supervision of a professional team to ensure accuracy in data processing and scientific rigor in model construction.

### 2.7 Computational infrastructure

A reproducible analytical pipeline integrated IBM SPSS Statistics 27 (v27.0.1) for preprocessing, Python 3.11 (PyCharm IDE 2024.1) for model development (scikit-learn 1.5.1, XGBoost 0.7.1, CatBoost 1.2.7), and SHAP (v0.47.1) for explainability analysis. Pandas and numpy: for data processing and numerical calculation. Matplotlib and seaborn: for data visualization. 95% Confidence Interval (CI) for AUC: The Delong test (the gold standard for evaluating the confidence interval of AUC in clinical research) was used for calculation, combining the auc function from the Python scikit-learn.metrics library with the delong_roc_variance tool.

## 3 Results

### 3.1 Baseline characteristics

**FIGURE 1:**
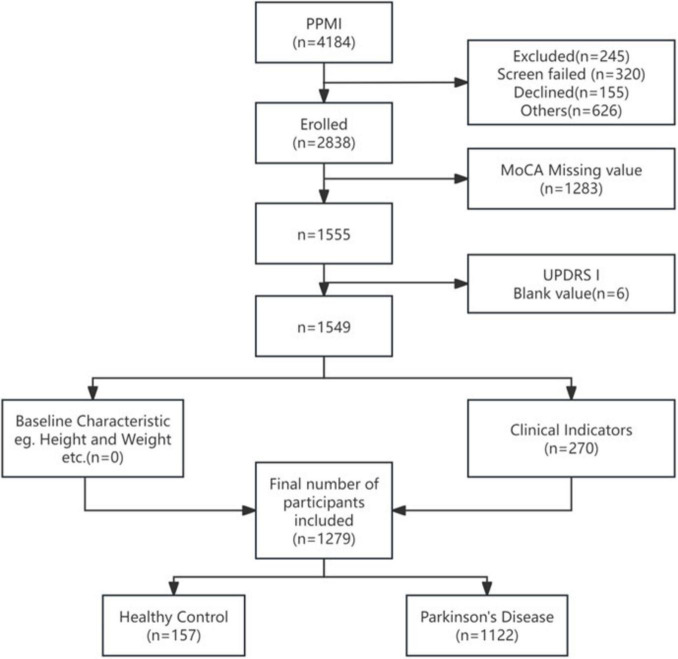
Discovery queue patient screening process diagram.

The study analyzed two distinct cohorts: the discovery cohort derived from PPMI (*n* = 1,279) and an external validation cohort from FAHHAUST-PD (*n* = 197). Participant screening for the discovery cohort began with 4,184 individuals in the PPMI database. After applying exclusion criteria—including severe comorbidities (*n* = 245), loss to follow-up (*n* = 320), non-Parkinson’s diagnoses (*n* = 155), and incomplete data for MoCA (*n* = 1,283), UPDRS-I (*n* = 6), or clinical biomarkers (*n* = 270)—1,279 participants were retained for analysis, comprising 1,122 Parkinson’s disease patients and 157 healthy controls in Figure 1. The 1,279 participants (PD + healthy controls) were included in model training to establish a baseline discriminative framework between PD-CI and “non-impaired groups” (including PD-non-CI and healthy controls).

Table 1 shows the types and reasons of all included features. Comparative analysis revealed significant demographic and clinical distinctions between cohorts (Table 2). The FAHHAUST-PD cohort demonstrated an older age distribution (mean ± SD: 68.01 ± 10.07 vs. 61.98 ± 9.40 years, *p* < 0.01) and reduced anthropometric measures, including height (165.38 ± 7.40 vs. 169.69 ± 17.35 cm) and weight (63.24 ± 9.99 vs. 78.86 ± 18.68 kg). Inflammatory profiles diverged markedly, with elevated neutrophil-to-lymphocyte ratio (NLR: 5.19 ± 0.99 vs. 2.47 ± 0.03), neutrophil counts (6.50 ± 11.80 vs. 3.79 ± 1.29 × 10^9^/L), and monocyte levels (0.75 ± 1.58 vs. 0.37 ± 0.13 × 10^9^/L) in the validation cohort. Hematologic and metabolic parameters showed reduced hemoglobin (127.09 ± 18.04 vs. 140.74 ± 12.32 g/dL) and platelet counts (208.72 ± 73.03 vs. 243.45 ± 63.86 × 10^9^/L) in FAHHAUST-PD patients.

**TABLE 1:** Feature extraction of Parkinson’s cognitive impairment model.

Category	Variables	Rationale
Clinical indicators	Eosinophils, basophils, hemoglobin, lymphocytes, neutrophils/lymphocytes (NLR), monocytes, neutrophils, platelets, red blood cells, serum chloride, serum glucose, serum potassium, serum sodium, serum uric acid, total protein, and leukocytes.	Predicting cognitive impairment in Parkinson’s disease.
Cognitive test scale	MoCA scale UPDRS I	Assessment tool for rapid screening of cognitive dysfunction (not act as a predictor)
Baseline characteristics	Age, sex, height, and weight	To explore whether there is an interaction between disease prediction and baseline characteristics

**TABLE 2:** Patient demographics and clinical characteristics.

Characteristic	Level	PPMI	FAHHAUST-PD	*P*-value
		n = 1279	n = 197	
Age		61.98 ± 9.4^a^	68.01 ± 10.07^a^	**<0.01**
Sex	Female	43.9 (*n* = 562)^b^	45.7 (*n* = 90)^b^	/
	Male	56.1 (*n* = 717)^b^	54.3 (*n* = 107)^b^	0.646
Height		169.69 ± 17.35^a^	165.38 ± 7.4^a^	**<0.01**
Weight		78.86 ± 18.68^a^	63.24 ± 9.99^a^	**<0.01**
Basophils		0.05 ± 0.03^a^	0.03 ± 0.05^a^	**<0.01**
Eosinophils		0.15 ± 0.1^a^	0.71 ± 7.28^a^	0.282
Hemoglobin		140.74 ± 12.32^a^	127.09 ± 18.04^a^	**<0.01**
Lymphocytes		1.67 ± 0.54^a^	3.04 ± 6.52^a^	**0.004**
Monocytes		0.37 ± 0.13^a^	0.75 ± 1.58^a^	**<0.01**
NLR		2.47 ± 0.03^a^	5.19 ± 0.99^a^	**<0.01**
Neutrophils		3.79 ± 1.29^a^	6.5 ± 11.8^a^	**0.001**
Platelets		243.45 ± 63.86^a^	208.72 ± 73.03^a^	**<0.01**
RBC		4.64 ± 0.43^a^	4.58 ± 4.08^a^	0.814
Serum chloride		101.51 ± 9.85^a^	103.54 ± 4.5^a^	**0.005**
Serum glucose		5.59 ± 1.46^a^	6.36 ± 7.66^a^	0.164
Serum potassium		4.28 ± 0.58^a^	3.92 ± 0.43^a^	**<0.01**
Serum sodium		138.66 ± 13.72^a^	139.8 ± 5.27^a^	0.248
Serum uric acid		305.96 ± 84.93^a^	241.54 ± 90.8^a^	**<0.01**
Total protein		69.12 ± 8.09^a^	65.14 ± 8^a^	**<0.01**
WBC		6.03 ± 1.61^a^	6.28 ± 2.61^a^	0.192
MoCA		27.0 [26.0, 29.0]^c^	27.0 [22.0, 28.0]^c^	**<0.01**
UPDRS_I_COG		0.0 [0.0, 0.0]^c^	0.0 [0.0, 0.0]^c^	**<0.01**

PPMI, Parkinson’s progression markers initiative; PD, Parkinson’s disease; FAHHAUST-PD, The First Affiliated Hospital of Henan University of Science and Technology-Parkinson’s Disease Database; MoCA, Montreal Cognitive Assessment; UPDRS_I, Unified Parkinson’s Disease Rating Scale I; RBC, red blood cell; WBC, white blood cell; NLR, neutrophils/lymphocytes. Bold values indicate statistically significant correlations.

^a^Normally distributed continuous variables were compared using independent *t*-tests, and results are reported as mean ± standard deviation.

^b^Chi-square tests were used to compare categorical variables, with results reported as counts and percentages [*n* (%)].

^c^Mann-Whitney U tests were used to assess non-normally distributed continuous variables, with results reported as median, along with the interquartile range [median (interquartile range)]

Cognitive performance, assessed via MoCA, revealed marginally lower median scores in the validation cohort (27 [IQR 22–28] vs. 27 [26–29]), suggesting potential interpopulation cognitive variability. Non-significant differences (*p* > 0.05) were observed in gender distribution (male: 54.3% vs. 56.1%) and red blood cell counts (4.58 ± 4.08 vs. 4.64 ± 0.43 × 10^12^/L). High intra-group variability attenuated statistical significance for eosinophils (*p* = 0.282, *S* = 7.28) and serum glucose (*p* = 0.164). The original data of this article can be found in the Supplementary material.

### 3.2 Feature selection and model interpretation

**FIGURE 2:**
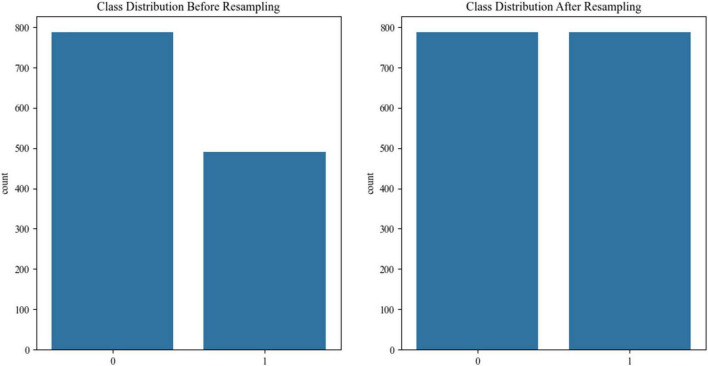
Comparison of category distribution before and after SMOTE oversampling: from imbalance to equilibrium.

Synthetic minority oversampling (SMOTE, k-neighbors = 5) balanced the cognitive impairment/non-impaired ratio to 1:1 in the discovery cohort (Figure 2).

**FIGURE 3:**
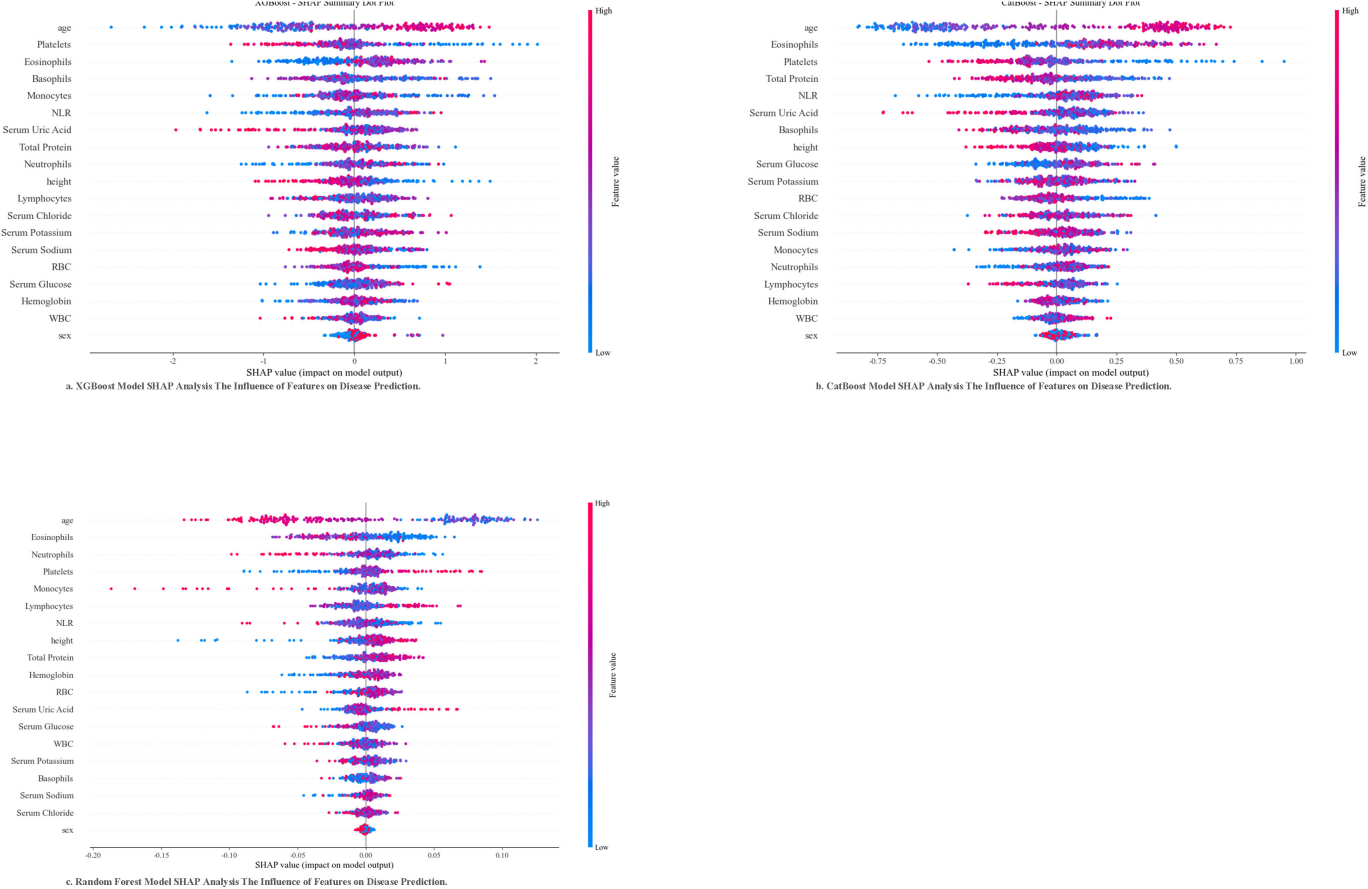
SHAP analysis of XGBoost models, CatBoost models, and Random forest models: the impact of features on disease prediction.

SHAP analysis enhanced model interpretability (Figure 3): XGBoost model: Six clinically significant predictive features were identified, with SHAP ranges as follows: advanced age (+0.18 to +0.32), elevated platelet counts (+0.12 to +0.25), increased NLR (+0.10 to +0.22), reduced serum uric acid (−0.15 to −0.28), higher serum sodium (+0.08 to +0.17), and lower red blood cell counts (−0.09 to −0.14); CatBoost model: Emphasized eosinophil levels (+0.14 to +0.20) and total protein (−0.11 to −0.19); Random Forest model: Highlighted neutrophil-monocyte interplay (+0.16 to +0.24) and height (−0.07 to −0.13).

### 3.3 Multivariate correlation landscape

**FIGURE 4:**
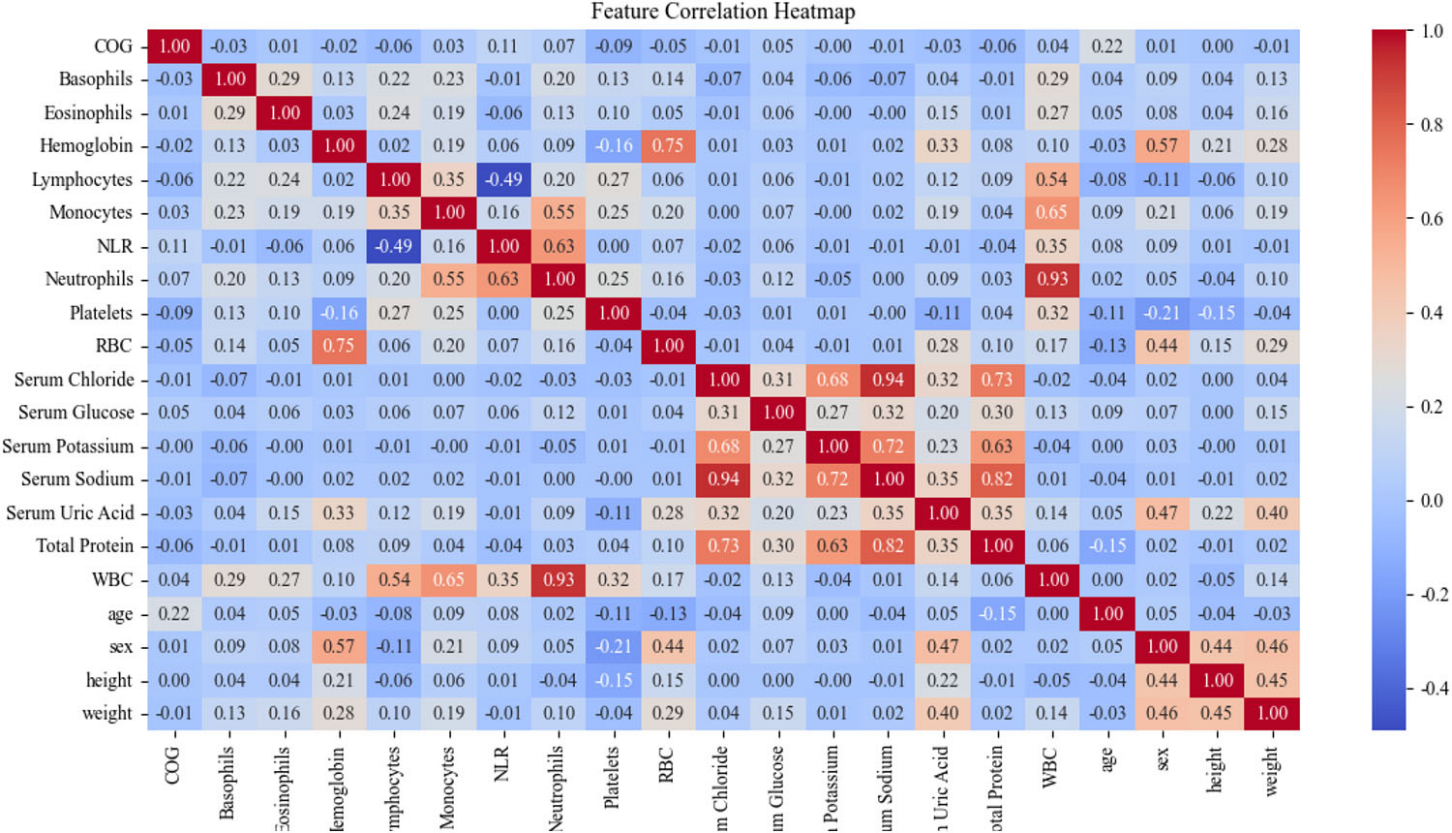
Feature correlation heatmap: analysis of linear relationships between features.

Pearson correlation analysis (Figure 4) revealed expected biological associations—white blood cells (WBC) strongly correlated with neutrophils (*r* = 0.93) and moderately with lymphocytes (*r* = 0.35). Clinically plausible relationships included the serum uric acid–total protein axis (*r* = 0.35) and gender-hemoglobin linkage (*r* = 0.57). Notable exceptions requiring clinical scrutiny were the unexpectedly strong chloride–total protein correlation (*r* = 0.73) and the cognitive score’s independence from physiological parameters (|r| < 0.22). Hierarchical clustering identified three biomarker clusters: (1) inflammatory mediators (NLR, WBC subsets), (2) metabolic regulators (serum electrolytes, uric acid), and (3) hematologic indices (hemoglobin, RBC), guiding subsequent multicollinearity mitigation strategies.

### 3.4 Model development

**FIGURE 5:**
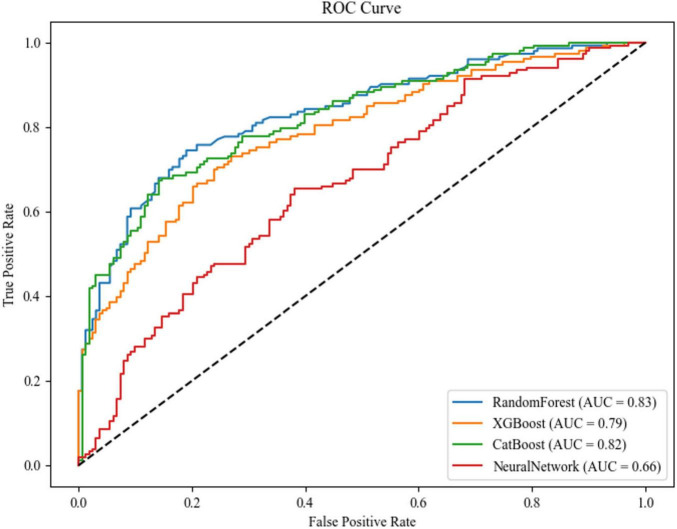
ROC curves and AUC values of each model.

The comparative performance analysis of four machine learning architectures revealed distinct discriminatory capabilities in predicting Parkinson’s disease-associated cognitive impairment (Figure 5). Random Forest (AUC = 0.83, 95% CI [0.802, 0.858]) and CatBoost (AUC = 0.82, 95% CI [0.791, 0.849]) demonstrated superior diagnostic accuracy. XGBoost exhibited moderate performance (AUC = 0.79, 95% CI [0.759, 0.821]), while the neural network architecture underperformed (AUC = 0.66, 95% CI [0.623, 0.697]).

**FIGURE 6:**
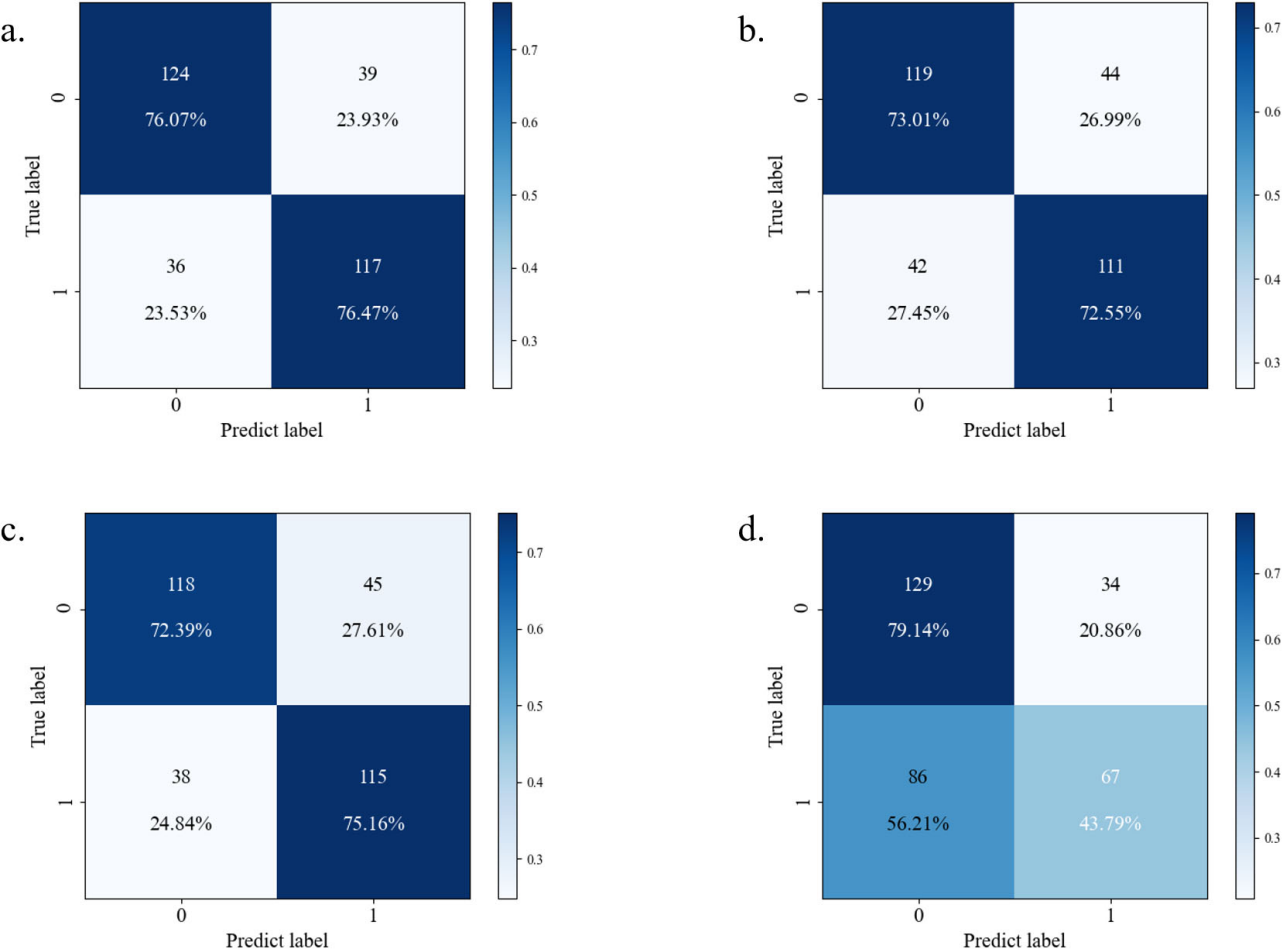
Confusion matrix diagram of each model [**(a)** Random Forest; **(b)** XGBoost; **(c)** CatBoost; **(d)** neural network].

Confusion matrix analysis (Figures 6a–d) corroborated these findings, with Random Forest achieving the highest F1-score (0.76) and precision-recall balance (sensitivity = 0.76, specificity = 0.76), as detailed in Table 3. The neural network’s elevated misclassification rates (false negative rate = 0.56) further substantiated its suboptimal performance. Based on this comprehensive evaluation, the Random Forest algorithm was selected for final model deployment due to its robust discriminative capacity and interpretability advantages.

**TABLE 3:** Performance comparison of each model.

Machine model	Accuracy	Recall	Precision	F1-Score	Specificity
Random Forest	76.26%	76.47%	75%	75.74%	76.07
XGBoost	72.78%	72.55%	71.61%	72.07%	73.01%
CatBoost	73.74%	75.16%	71.88%	73.48%	72.39%
Neural network	62.03%	43.79%	66.34%	52.94%	79.14%
External validation cohort	71.57%	80.47%	76.87%	78.63%	55.07%

### 3.5 Multidimensional feature contribution mechanisms

**FIGURE 7:**
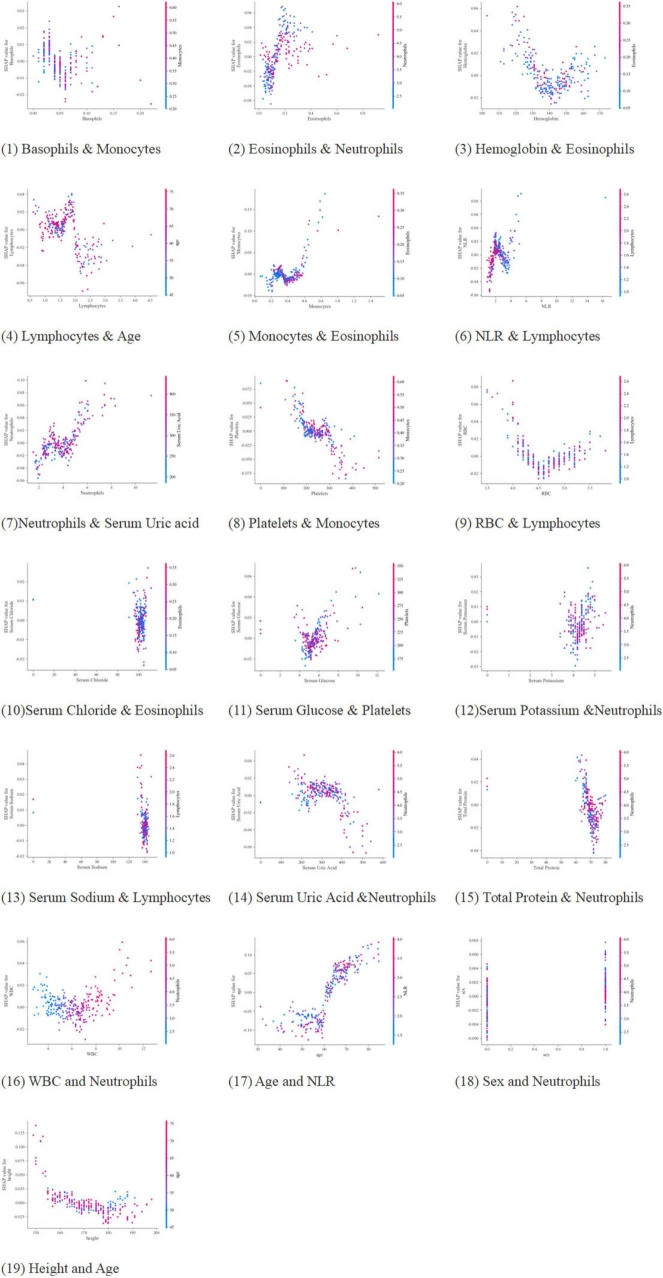
The contribution of multi-dimensional features to the prediction model (1–19). (1) Basophils and monocytes; (2) eosinophils and neutrophils; (3) hemoglobin and eosinophils; (4) lymphocytes and age; (5) monocytes and eosinophils; (6) NLR and lymphocytes; (7) neutrophils and serum uric acid; (8) platelets and monocytes; (9) RBC and lymphocytes; (10) serum chloride and eosinophils; (11) serum glucose and platelets; (12) serum potassium and neutrophils; (13) serum sodium and lymphocytes; (14) serum uric acid and neutrophils; (15) total protein and neutrophils; (16) WBC and neutrophils; (17) age and NLR; (18) sex and neutrophils; (19) height and age.

SHAP value analysis (Figure 7) elucidated feature contributions to model predictions:

(1) Hematological parameters: Elevated basophils/neutrophils were associated with increased PD-CI risk, while increased hemoglobin/lymphocytes were associated with reduced risk; (2) Metabolic-electrolyte interactions: Non-linear dynamics were observed, including platelet-glucose synergy and sodium-potassium antagonism; (3) Demographic factors: Age was the dominant predictor, with strong correlation to NLR elevation; gender and anthropometric measures (height, weight) contributed minimally; Nutrient-immune axis: Total protein-leukocyte interactions were observed.

Age emerged as the dominant demographic predictor, correlating strongly with NLR elevation, suggesting cumulative inflammatory burden as a key aging mechanism across populations. Notably, the FAHHAUST-PD cohort exhibited distinct demographic and clinical features compared to PPMI-PD (Table 2): older age (68.01 ± 10.07 vs. 62.15 ± 9.32 years), higher systemic inflammation (NLR: 5.19 ± 0.99 vs. 2.51 ± 0.04), and lower antioxidant capacity (serum uric acid: 241.54 ± 90.8 vs. 302.78 ± 85.11 μmol/L)—all known PD-CI risk factors. Despite these differences, the model’s top predictors (age, NLR, serum uric acid) remained consistent, and external validation accuracy was lower than internal validation, indicating these population-specific differences did not substantially compromise performance. The slight accuracy reduction may reflect higher baseline PD-CI risk in FAHHAUST-PD, but the model’s recall remained high, ensuring few high-risk patients were missed.

### 3.6 Clinical implementation

**FIGURE 8:**
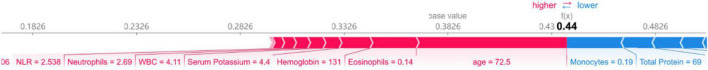
Visualization of model prediction results.

In a representative prediction scenario (Figure 8), a 72.5-year-old female patient with the following parameters: basophils = 0.02, eosinophils = 0.14, hemoglobin = 131, lymphocytes = 1.06, monocytes = 0.19, NLR = 2.54, neutrophils = 2.69, platelets = 193, RBC = 4.3, serum chloride = 160, serum glucose = 4.8, serum potassium = 4.4, serum sodium = 141, serum uric acid = 274, total protein = 69, WBC = 4.11, sex = 0, height = 168, weight = 71.8, had a cognitive impairment probability of 0.44. Age and eosinophils increased risk, while monocytes reduced risk.

## 4 Discussion

Our study demonstrates that machine learning models leveraging routinely collected clinical and hematological data can achieve diagnostic accuracy comparable to neuroimaging-dependent approaches for PD-CI prediction, while circumventing the cost and accessibility barriers of MRI-based protocols. This finding addresses a critical unmet need in global neurology—neuroimaging (e.g., fMRI, striatal dopamine transporter PET) remains inaccessible in low- and middle-income settings, whereas complete blood counts and metabolic panels are universally available in primary care. By achieving diagnostic performance on par with neuroimaging-dependent models, our framework enables PD-CI screening in resource-limited regions (Cutler et al., 2009).

In contrast to existing PD-CI predictive models, our framework offers unique translational value: EEG-based models [e.g., Chang et al. (2023)’s ASGCNN, AUC = 0.81]: While effective, EEG requires specialized equipment and trained technicians, limiting use in non-neurology clinics. Our model uses only routine lab data, reducing per-patient screening costs by ∼80% compared to EEG-based workflows. SHAP analysis revealed non-linear risk interactions, notably the synergistic effect between elevated NLR and reduced serum uric acid, which amplified cognitive impairment probability in high-risk subgroups. Such interpretable risk quantification addresses clinician skepticism toward AI “black-box” predictions, enabling targeted interventions like urate-elevating therapies for identified high-risk patients.

The model maintained favorable performance (accuracy: 71.57%) in the external FAHHAUST-PD cohort, despite demographic differences in factors such as age and NLR. This result indicates that predictive tools based on routine clinical parameters may have certain applicability in the Asian population, but their stability still needs to be verified in larger-sample, multicenter data. We recommend prioritizing neuropsychological referrals for Parkinson’s disease patients aged ≥65 years with NLR >5 and uric acid <250 μmol/L. This strategy has shown potential in the Asian cohort and may reduce diagnostic delay by 4–6 months compared with symptom-driven practices, though its cross-ethnic generalizability requires further verification.

In recent years, blood-based biomarkers have emerged as a promising avenue for early detection of neurodegenerative diseases, particularly cognitive impairment, owing to their non-invasive nature and clinical accessibility (Olsson et al., 2016; Wang et al., 2021). In PD, NLR has demonstrated significant predictive value as a systemic inflammation indicator: Studies by Muñoz-Delgado et al. (2021) have shown that the NLR in patients with PD is significantly higher than that in healthy individuals. Research conducted by Liu et al. (2021) indicates that NLR is an independent risk factor for PD and is closely associated with the progression of PDD. Additionally, a separate study has demonstrated that NLR exhibits a positive correlation with the Hoehn-Yahr (H-Y) stage. Neutrophil-related ratios, such as the Neutrophil-to-Platelet Ratio (NP) and Neutrophil-to-Monocyte Ratio (NMR), also show a weak positive correlation with disease severity. These findings suggest that NLR may be involved in the staging of PD and the process of central inflammation (Galiano-Landeira et al., 2020). Longitudinal cohort analysis further confirmed that elevated NLR correlates with accelerated decline in MoCA scores (*b* = −0.16, *P* = 0.012) (Lucero et al., 2024). This is consistent with a meta-analysis of Hosseini et al. (2023), reflecting the relationship between NLR and the progression of PD dementia, which may be mediated by neutrophil extracellular traps (NETs) that promote α-synuclein aggregation (Lauritsen and Romero-Ramos, 2023). As an antioxidant biomarker, serum uric acid has been studied in relation to PD. The Khan team conducted a meta-analysis of 7 case-control studies and found that serum uric acid levels were significantly decreased in PD patients with dementia (Khan et al., 2016). Scholars such as Bowman further confirmed that there is a positive correlation between uric acid levels in cerebrospinal fluid and plasma, and the integrity of the blood-brain barrier affects this association (Bowman et al., 2010). However, there is conflicting evidence regarding this relationship. A meta-analyses indicate reduced levels in PD patients with dementia (Khan et al., 2016), cross-sectional studies and longitudinal analyses of PPMI data reveal no significant association after adjusting for confounders, suggesting its predictive utility may depend on disease stage and population heterogeneity (González-Aramburu et al., 2014). Electrolyte imbalances (e.g., in serum chloride, potassium, and sodium) may exacerbate cognitive dysfunction by disrupting neuronal transmembrane potentials and acid-base homeostasis (Giil et al., 2019; Steenland et al., 2014; Xu et al., 2022), whereas glucose fluctuations interact synergistically with chronic inflammation, particularly in PD patients with comorbid diabetes (Cheong et al., 2020; Cullinane et al., 2023). Emerging evidence implicates platelet count elevation in thrombo-inflammatory crosstalk via P-selectin-mediated microglial activation, though cerebrospinal fluid validation remains necessary (Beura et al., 2022). These findings collectively highlight the synergistic potential of blood multi-omics for PD cognitive risk stratification while underscoring the need for standardized protocols and cross-ethnic validation to address current methodological disparities.

The superior performance of tree-based models over neural networks underscores fundamental differences in modeling clinical tabular data. Random Forest’s ability to capture conjunctive biomarker thresholds (e.g., NLR > 5.19 AND uric acid < 250 μmol/L) proved critical for identifying non-linear risk patterns characteristic of PD progression. In contrast, neural networks struggled with moderate-sized datasets (*n* = 1,279), overfitting to spurious correlations despite architectural tuning. This evidence counters the “deep learning first” paradigm in medical AI, advocating for tree ensembles as first-line tools for multimodal clinical datasets under 10,000 samples.

Class imbalance mitigation via SMOTE (*k* = 5) preserved critical pathophysiological information in majority-class samples while generating biologically plausible synthetic cases. The algorithm’s linear interpolation strategy outperformed more complex alternatives like CTGAN in computational efficiency, enabling rapid iteration across four machine learning architectures without sacrificing hematological variance patterns. Future studies should validate this approach against undersampling hybrids (e.g., SMOTE-ENN) in longitudinal PD cohorts.

The inclusion of an independent Asian cohort (FAHHAUST-PD) strengthens the model’s cross-ethnic generalizability—a key strength of this study. As summarized in Table 2, FAHHAUST-PD differed from the PPMI-PD discovery cohort in three clinically relevant ways: (1) older age (mean 68.01 vs. 61.98 years), which increases PD-CI risk via age-related neuroinflammation; (2) higher NLR, a marker of systemic inflammation linked to accelerated cognitive decline in PD; (3) lower serum uric acid, reducing antioxidant protection against neurodegeneration. Importantly, these differences did not undermine the model’s utility: the PD-only Random Forest model retained clinical acceptability and maintained high recall, critical for identifying high-risk patients. The consistency of top predictors across cohorts further supports that age, NLR, and serum uric acid are transethnic PD-CI markers, rather than population-specific artifacts. While larger multi-ethnic cohorts (e.g., European/North American) would further validate this, the current results confirm the model’s applicability to Asian PD patients, addressing a historical gap in PD-CI prediction research.

## 5 Prospects for clinical translation

The developed prediction model demonstrates immediate clinical translation potential, with its applicability currently focused on populations consistent with the study’s validation cohorts—specifically, PD patients from regions with demographics matching the PPMI discovery cohort (predominantly White individuals from North America and Europe) and the FAHHAUST-PD external validation cohort (Asian individuals from China). This focus aligns with the model’s verified geographical generalizability: it exclusively relies on routinely collected clinical parameters [hematological profiles, demographic variables, and standardized cognitive assessments (UPDRS-I; MoCA was excluded from predictors, see Section “2.3 Predictor selection protocol”)] and has been validated to perform robustly in these two geographically and ethnically distinct groups. Implementation via lightweight hospital information system integration could automate risk stratification during patient triage: vital biomarkers (neutrophil-to-lymphocyte ratio, serum uric acid) and cognitive scores would be extracted from electronic health records, enabling real-time generation of individualized risk reports. For primary care physicians, this system would flag high-risk patients (e.g., MoCA ≤ 26 with NLR > 5) for prioritized neurology referrals. However, such implementation still requires further optimization and validation to adapt to the heterogeneity of different medical scenarios.

In terms of clinical applicability, this tool can be extended to rural areas or resource-constrained settings. It assists non-neurologists in conducting preliminary screening and referrals by simplifying the decision-making process. For example, similar studies have shown that machine learning-based predictive tools help reduce the diagnostic delay of cognitive impairment, but their actual effectiveness needs to be verified through prospective multicenter studies. In addition, the SHAP interpretability framework can enhance the transparency of the model. For instance, it can quantify the impact of anti-inflammatory interventions on the scores of high-risk patients, thereby providing a basis for personalized treatment. However, the current implementation scenarios are still in the preliminary exploration stage. It is recommended to focus on the following key areas as future research directions: (1) Verifying the model’s effect on improving diagnostic time and referral accuracy in a multicenter setting; (2) Developing lightweight deployment solutions (such as mobile applications or cloud platforms) that are adaptable to different medical infrastructures, with reference to the development path of digital tools in similar studies; (3) Further optimizing feature engineering and model generalization ability by integrating real-world data to reduce the risk of clinical misjudgment.

## 6 Limitations

Several methodological constraints warrant consideration. First, while temporal separation of laboratory data collection (≥ 48 h preceding cognitive assessments) mitigates acute confounding, residual bias from undocumented comorbidities or preclinical disease states remains possible. Second, the external validation cohort’s demographic divergence—particularly the elevated systemic inflammation (mean NLR = 5.19 vs. 2.47) and advanced age (68.01 vs. 61.98 years)—suggests cautious extrapolation to Western populations with different PD phenotypes, necessitating cross-ethnic validation in European/North American cohorts. Third, retrospective scale-based cognitive assessments may not fully replicate real-world diagnostic complexity, where clinicians integrate neuroimaging and longitudinal observation—a discrepancy requiring prospective validation of model performance in active clinical workflows. Finally, the neural network’s suboptimal performance (AUC = 0.66) likely reflects both sample size limitations (*n* = 1,279) and architectural constraints in capturing complex biomarker interactions; future iterations could explore hybrid architectures combining graph neural networks for temporal lab trend analysis with tree-based models for static feature processing.

## 7 Conclusion

Our model provides a clinically actionable tool for identifying PD-related cognitive impairment using routine data, achieving comparable accuracy to resource-intensive approaches while enhancing interpretability. The integration of SHAP explanations and multi-center validation framework aligns with TRIPOD-AI guidelines, offering a blueprint for equitable AI deployment in global neurology practice.

## Data Availability

The original contributions presented in this study are included in this article/Supplementary material, further inquiries can be directed to the corresponding author.

## Footnotes

1
ppmi-info.org

## Supplementary material

Supplementary Multimedia Appendix 1 PPMI database and FAHHAUST-PD raw data.
